# Assessing ectomycorrhizal associations and transgene expression in transgenic *Castanea dentata*

**DOI:** 10.1186/1753-6561-5-S7-O54

**Published:** 2011-09-13

**Authors:** Katherine D'Amico, Thomas Horton, Charles Maynard, William Powell

**Affiliations:** 1 State University of New York College of Environmental Science and Forestry, USA

## Background

American chestnut (*Castanea dentata*) once dominated the forests of the eastern United States until the introduction of a Chinese fungal pathogen *Cryphonectria parasitica*(causal agent of the chestnut blight) decimated the species. This tree was important not only ecologically, but also played a significant role economically. Restoration programs are implementing a variety of techniques, including genetic transformation, to develop a blight resistant tree that can be reintroduced into the native chestnut range (S.A. Merkle, 2007, *Tree Genetics and Genomics*, 3, 111-18).

Specific genes involved in plant defense responses are being introduced into American chestnut via *Agrobacterium*-mediated transformation, with the hope that one gene or a combination of genes will aid in chestnut defense against blight (L.D. Polin, 2006, *Plant Cell*, *Tissue*, *and Organ Culture*, 84, 69-79). One gene of particular interest to the transgenic project is oxalate oxidase, a defense related gene from wheat that has also been shown to enhance the defense response of other plants when introduced through genetic transformation (H. Liang, 2001, *Plant Molecular Biology*, 45, 619-29). American chestnut transformation with a binary vector containing oxalate oxidase has produced a number of transgenic events, many of which have already been regenerated plants (unpublished data).

Before releasing any transgenic plant it is necessary to assess any non-target impacts the introduced genes may have on associated microbial communities. *C. parasitica*is a fungal pathogen, so we are particularly interested in studying the effects of any transgene on the symbiotic relationship between the host plant and mycorrhizae-forming fungi. Prior studies have examined the potential impact of transgenic plants on ectomycorrhizal fungi (K. L. Oliver, 2008, *Applied and Environmental Microbiology*, 74, 5340-48).

## Objectives

1. Analyze and compare transgene expression in root, stem, and leaf tissue in American chestnut trees transformed with a gene for oxalate oxidase.

2. Assess potential non-target impacts on mycorrhizal fungi by comparing ectomycorrhizal associations with wildtype American chestnut and a transgenic American chestnut expressing a gene for oxalate oxidase.

## Methods

Wildtype American chestnut seeds and transgenic American chestnut (‘Darling4’) trees were grown in a potting mix containing field soil in a soil bioassay to bait for mycorrhizal fungi. All trees were grown in a greenhouse for at least one year before mycorrhizal root tips were harvested. The root system of each tree was rinsed over a fine sieve, and segments of root were collected. Mycorrhizal root tips were quantified and grouped based on morphotype. Fungi were identified using RFLP and sequence analysis of the fungal ITS region.

RNA was extracted for gene expression studies from all tissues using a CTAB extraction method. RNA samples were then DNase treated and used to synthesize cDNA, which was used as a template in all subsequent quantitative RT-PCR experiments. qRT-PCR studies were conducted to determine the relative level of transgene expression in leaf, stem, and root tissue in ‘Darling4’ trees.

## Results

Gene expression study results based on qRT-PCR indicate that the oxalate oxidase gene is expressed in root tissue of ‘Darling4’ American chestnut and not in the wildtype control (Fig. 1). Transgene expression results for both stem and leaf tissue are comparable to that found in root tissues.

Based on mycorrhizal tip quantification for transgenic and wildtype American chestnut, it appears that there is not a significant difference in total percent fungal colonization between these tree types (Fig. 2).

## Discussion and conclusions

The results of the gene expression portion of this study show oxalate oxidase is expressed in the root tissue of ‘Darling4’ transgenic American chestnut trees. As expected, there is no oxalate oxidase expression in root tissue of wildtype American chestnut trees. Oxalate oxidase expression in root tissue has the potential to influence associated microbial communities, specifically mycorrhizal fungi that are in direct contact with chestnut roots. It is therefore necessary to assess mycorrhizal associations in transgenic chestnut to determine if there are any non-target impacts as a result of this expression. Mycorrhizal root tips were quantified for both the Darling 4 transgenic chestnut and the wildtype. The results of the total percent ectomycorrhizal fungal colonization portion of the study show that there is no significant difference in overall levels of mycorrhizal abundance between the two tree types. It can be inferred from this that American chestnut trees expressing oxalate oxidase are able to form mycorrhizal associations in a similar manner to the wildtype.

As with any other transgenic plant, ‘Darling4’ and other transgenic American chestnut trees are required to go through extensive deregulation procedures before they can be released back to the native chestnut range. The results from this study will be used in conjunction with other ecological impact studies to inform the deregulation process in an effort to restore the American chestnut. This study will also contribute to a body of existing work examining the potential environmental impacts of transgenic forest trees that can inform future policy and procedures relating to the genetic modification of important species.

